# The Development of Four-Arm PEG-Based Thermoresponsive Dexamethasone Prodrugs for the Treatment of Osteoarthritis Pain

**DOI:** 10.3390/nano16140893

**Published:** 2026-07-21

**Authors:** Yangwei Deng, Shabnam Arash, Jie Rong, Salma Abdullah Althobaiti, Shanshan Liu, Eleanor Ransdell-Green, Edward V. Fehringer, Dong Wang

**Affiliations:** 1 Department of Pharmaceutical Sciences, College of Pharmacy, University of Nebraska Medical Center, Omaha, NE 68198-6125, USA; xavierahz1990@gmail.com (Y.D.); sarash@unmc.edu (S.A.); jrong@unmc.edu (J.R.); salthobaiti@unmc.edu (S.A.A.); shaliu@unmc.edu (S.L.); eransdellgreen@unmc.edu (E.R.-G.); 2 Department of Orthopaedic Surgery and Rehabilitation, College of Medicine, University of Nebraska Medical Center, Omaha, NE 68198-6125, USA; evfehringer@unmc.edu

**Keywords:** polymeric prodrug, hydrogel, osteoarthritis

## Abstract

Thermoresponsive polymeric prodrugs represent a promising strategy for localized and sustained in vivo drug delivery. In this work, two polyethylene glycol (PEG)-based dexamethasone (Dex) prodrugs with different Dex contents were synthesized using a four-arm PEG scaffold. Prodrug **1**, containing four Dex molecules, showed high aqueous solubility but no thermoresponsive gelation behavior. In contrast, eight-Dex Prodrug **2** exhibited temperature-dependent aggregation and formed hydrogels in aqueous media. The viscosity of the Prodrug **2** hydrogel was reduced by introducing 10% ethanol as a cosolvent, enabling an injectable formulation that rapidly forms a hydrogel depot upon contact with aqueous media. The hydrogel provides gradual Dex release via the cleavage of the acid-labile hydrazone bond linking Dex to the PEG. In a monosodium iodoacetate (MIA)-induced osteoarthritis (OA) pain model, intra-articular (IA) injection of Prodrug **2** produced rapid and sustained pain relief for up to 28 days. These findings indicate that the hydrogel-forming four-arm PEG-based Dex prodrug offers a potentially effective approach for prolonged local corticosteroid (CS) delivery, applicable to the treatment of many local pathologies, including OA and OA pain.

## 1. Introduction

Osteoarthritis (OA) is the most prevalent chronic joint disease, and a major cause of pain and disability worldwide. In the United States alone, it is estimated to affect more than 30 million individuals [1,2,3]. The disease is characterized by progressive degeneration of articular cartilage, pathological remodeling of subchondral bone, and chronic inflammation of the synovial joint, which together lead to persistent pain and restricted mobility [4]. Currently, no approved therapies can halt or reverse the structural progression of OA, and clinical management primarily focuses on alleviating pain and inflammation [5,6,7]. Among available treatment options, intra-articular (IA) corticosteroid (CS) injections remain one of the most widely used interventions for OA-associated joint pain [8,9,10]. However, the short duration of IA CS, generally lasting only a few weeks, has often limited its therapeutic benefits. Therefore, repeated injections are necessary, which may increase systemic exposure and the risk of diverse CS side effects and injection-related infections [11,12,13].

To address these limitations, considerable research has focused on developing long-acting local drug delivery systems capable of sustaining CS activity within the joint cavity. Zilretta^®^, an intra-articular poly(lactic-co-glycolic acid) (PLGA) microsphere-based triamcinolone acetonide formulation [14], was approved by FDA in 2017 for clinical management of knee OA pain. Despite this approval, its commercial uptake has been limited, with projected sales reaching only $117 million in 2025, representing 0.9% of the OA therapeutic market [15]. TLC599, a liposomal dexamethasone phosphate sodium salt formulation, is currently being tested in clinical trials [16]. Injectable hydrogels and polymer-based drug carriers have emerged as promising approaches for improving drug retention and supporting controlled release of therapeutics in arthritic joints and other inflammatory sites [17,18,19,20,21,22]. In particular, thermoresponsive hydrogels that undergo sol–gel transition at physiological temperatures have gained significant interest, as they can be administered as low-viscosity solutions and subsequently form a depot at the injection site [23,24,25]. Such in situ forming systems can extend drug retention time in the synovial cavity and maintain therapeutic drug levels locally, consequently reducing dosing frequency and minimizing systemic adverse effects [26].

Polymeric prodrug strategies offer an additional approach to achieving sustained and localized drug release. By covalently attaching therapeutic agents to polymeric carriers through stimuli-responsive linker mechanisms, drug activation can be regulated under specific pathophysiological conditions, supporting more precisely controlled drug activation/release at pathological sites [23,25,27]. In particular, we have reported the development of ProGel-Dex, a *N*-(2-hydroxypropyl) methacrylamide (HPMA) copolymer-based Dex prodrug in which Dex is conjugated to the polymeric carrier through an acid-labile hydrazone linker, allowing intracellular activation of the prodrug in the acidic compartments of inflammatory cells [23,28,29,30]. Studies have shown that increasing the hydrophobic drug content of Dex polymeric prodrugs can induce thermoresponsive behavior, allowing the formation of injectable hydrogels that provide sustained local analgesic and anti-inflammatory effects in rodent arthritis models [25]. Notably, given its prodrug nature, the drug loading in ProGel-Dex formulation is significantly higher than the microsphere and liposomal CS formulations, resulting in a reduced injection volume at equivalent doses. One limitation of ProGel-Dex formulation, however, is its potential batch-to-batch variation in molecular weight and Dex loading, due to the free radical nature of the Reversible Addition-Fragmentation chain Transfer (RAFT) polymerization. While through proper chemical engineering strategies, this issue may be mitigated during the Chemistry, Manufacturing, and Controls (CMC) development, there remains a need to develop polymeric prodrug designs with more definitive chemical structures, to fundamentally address this issue.

In the present study, we developed four-arm polyethylene glycol (PEG)-based Dex prodrugs with tunable drug densities and investigated their thermoresponsive behavior and prospects as IA treatment for sustained OA pain management. Two polymeric prodrugs were synthesized using a 5 kDa four-arm PEG scaffold, yielding Prodrug **1** containing four Dex and Prodrug **2** containing eight Dex. The influence of drug loading on polymer solubility, thermoresponsive aggregation, and hydrogel formation was systematically examined. Prodrug **1** remained highly soluble without gelation at all tested temperature ranges (from 28 °C to 50 °C), whereas Prodrug **2** exhibited pronounced thermoresponsive behavior and formed hydrogels in aqueous media. To enable IA administration, a cosolvent-based formulation strategy was employed to reduce prodrug viscosity and produce an injectable system capable of forming a prodrug depot upon exposure to in vivo environments. The resulting formulation was further characterized and tested for therapeutic efficacy in a monosodium iodoacetate (MIA)-induced OA pain mouse model.

## 2. Materials and Methods

**Materials**: Dexamethasone was acquired from Tianjin Pharmaceuticals Group Co., Ltd. (Tianjin, China). Dexamethasone 21-phosphate disodium was purchased from Sigma-Aldrich (Milwaukee, WI, USA). Four-arm PEG (X-PEG5k-COOH) was obtained from Creative PEGWorks (Chapel Hill, NC, USA). Sephadex LH-20 resin was purchased from GE Healthcare (Piscataway, NJ, USA). Unless otherwise specified, all solvents and reagents were purchased from Fisher Scientific, Sigma-Aldrich, or ACROS, and used without further purification.

**Instruments**: ^1^H NMR spectra were recorded on a 400 MHz NMR spectrometer (Bruker Spatial Biology, Inc., Bothell, WA, USA). The molecular weights of polymeric prodrugs were determined using a gel permeation chromatography (GPC) system equipped with a PLgel 5 μm MIXED-C column (Agilent Technologies, Santa Clara, CA, USA), a DAWN 8+ multiangle light scattering (MALS) system (Wyatt Technology Corporation, Santa Barbara, CA, USA) and an Optilab T-rEX refractive index detector (Wyatt). GPC was performed in the mobile phase of 0.01 wt% LiBr/DMF. The Dex contents of the macromolecular prodrugs were analyzed (after overnight hydrolysis at room temperature in 0.2 N HCl) on an Agilent 1260 Infinity II HPLC system (Agilent Technologies, Inc., Santa Clara, CA, USA) with a reverse phase C_18_ column (Agilent, 4.6 × 250 mm, 5 µm). The average hydrodynamic diameter and polydispersity index (PDI) of the micelles were determined by DLS experiments using a Zetasizer Nano ZS90 (Malvern Instruments, Worcestershire, UK). For pain analysis, Librae Incapacitance Tester for Mice from Ugo Basile S.R.L. (Gemonio VA, Italy) was used.

**Synthesis**: The synthesis of Dex- and hydrazone-containing intermediates (Compound **4** and Compound **6**) was carried out following previously reported procedures [31]. Briefly, dexamethasone was first protected with a *tert*-butyldimethylsilyl (TBDMS) group to yield a silylated intermediate, which was subsequently converted to the corresponding hydrazide through reaction with hydrazine. The hydrazide intermediate was then coupled with Fmoc-protected glycine via DCC-mediated condensation, followed by Fmoc deprotection to afford Compound **4**, a hydrazone-containing Dex derivative. For the synthesis of Compound **6**, Compound **4** was further reacted with Fmoc-protected glutamic acid using DCC/HOBt coupling to introduce a second Dex moiety. After subsequent Fmoc deprotection, the resulting Dex dimer was obtained as Compound **6**. All intermediates and final products were purified by column chromatography and characterized according to the previously reported methods.

Prodrug **1**. DCC (80.5 mg, 0.39 mmol), 1-hydroxybenzotriazole (HOBt, 59.7 mg, 0.39 mmol), and X-PEG5k-COOH (200 mg, 0.039 mmol) were dissolved in anhydrous *N*,*N*-Dimethylformamide (DMF, 3 mL) and stirred at room temperature for 1 h. Compound **4** (135 mg, 0.234 mmol) was then added, and the reaction was stirred for 24 h. The crude product was purified using a Sephadex LH-20 column to collect the polymer fraction. After solvent removal, the residue was dissolved in tetrahydrofuran (THF), followed by sequential addition of pyridine and hydrofluoric acid (HF)/pyridine. The reaction mixture was stirred overnight at room temperature to remove TBDMS protecting groups. The product was further purified by LH-20 column, dissolved in water and dioxane (10:1), and lyophilized to yield Prodrug **1** (206 mg, 76.4%). ^1^H NMR (500 MHz, CDCl_3_): δ (ppm) 10.16 (s, 0.56H), 10.08 (s, 1.07H), 9.20 (s, 1.02H), 9.02 (s, 1.53H), 8.09 (s, 1.58H), 7.60 (s, 3.18H), 6.86 (m, 0.97H), 6.77 (m, 0.71H), 6.63–6.73 (m, 2.24H), 6.52 (m, 1.88H), 6.45 (d, J = 12.0 Hz, 1.88H), 6.34 (m, 2.13H), 6.05 (d, J = 16.4 Hz, 1.85H), 5.83 (s, 1.08H), 5.74 (s, 0.55H), 4.84–5.11 (m, 8.68H), 4.41–4.54 (m, 5.52H), 4.28–4.40 (m, 6.40H), 4.03–4.16 (m, 10.56H), 3.83 (m, 4.83H), 3.66 (m, 454.00H), 3.37–3.51 (m, 12.32H), 3.11 (m, 5.34H), 2.52–2.72 (m, 7.64H), 2.26–2.46 (m, 19.61H), 2.13–2.25 (m, 14.89H), 1.70–1.84 (m, 9.23H), 1.58–1.68 (m, 4.71H), 1.51 (m, 15.66H), 1.13–1.36 (m, 8.27H), 0.97–1.09 (m, 11.69H), 0.88–0.96 (m, 11.81H), 0.76–0.88 (m, 4.03H).

Prodrug **2**. Prodrug **2** was synthesized using a similar procedure. DCC (80.5 mg, 0.39 mmol), HOBt (59.7 mg, 0.39 mmol), and X-PEG5k-COOH (200 mg, 0.039 mmol) were dissolved in anhydrous DMF (3 mL) and stirred for 1 h. Compound **6** (296 mg, 0.234 mmol) was then added, and the reaction was stirred for 48 h. After LH-20 purification, the residue was subjected to THF dissolution and HF–pyridine deprotection as described above. The product was further purified by LH-20 column, dissolved in water and dioxane (10:1), and lyophilized to yield Prodrug **2** (256 mg, 71.3%). ^1^H NMR (400 MHz, DMSO-d_6_): δ (ppm) 10.94 (d, J = 16.0, 1.51H), 10.85 (d, J = 12.0, 2.57H), 10.59 (m, 2.57H), 8.40 (s, 1.34H), 8.21 (s, 2.16H), 8.14 (m, 1.48H), 7.97 (m, 2.39H), 7.67–7.80 (m, 3.61H), 7.04 (d, J = 8.0 Hz, 1.79H), 6.89–6.96 (m, 0.91H), 6.80 (s, 2.95H), 6.56–6.73 (m, 4.62H), 6.39–6.52 (m, 4.78H), 6.18–6.32 (m, 4.90H), 6.00 (d, J = 19.3, 2.74H), 5.14 (m, 7.20H), 4.94 (s, 6.94H), 4.67 (m, 7.54H), 4.53 (d, J = 6.0 Hz, 3.58H), 4.48 (d, J = 5.9 Hz, 4.32H), 4.42 (m, 2.08H), 4.36 (m, 1.88H), 4.07–4.27 (m, 20.30H), 4.06 (d, J = 5.4 Hz, 4.03H), 3.94 (s, 8.72H), 3.78–3.90 (m, 4.27H), 3.69 (m, 2.79H), 3.56–3.66 (m, 16.40H), 3.52 (m, 454.00H), 2.95 (m, 10.61H), 2.56–2.72 (m, 6.73H), 2.06–2.39 (m, 40.11H), 1.96 (m, 4.76H), 1.86 (m, 4.98H), 1.65–1.79 (m, 10.15H), 1.60 (m, 7.91H), 1.43 (m, 30.37H), 1.34 (m, 10.08H), 1.07 (m, 9.18H), 0.86 (m, 23.2H), 0.79 (d, J = 7.33, 22.2H).

**Drug content measurement**: Polymeric prodrug samples (2 mg/mL) were dissolved in 0.2 N HCl containing 50% methanol in a 20 mL vial equipped with a magnetic stir bar. The vial was sealed and the solution was stirred at room temperature overnight to ensure complete hydrolysis. The solution was then neutralized with 0.2 N NaOH (in 50% methanol) and analyzed using an Agilent 1260 Infinity II HPLC system equipped with a C18 column (4.6 × 250 mm, 2.7 μm). The mobile phase consisted of acetonitrile/water (4:6), with UV detection at 240 nm, a flow rate of 0.5 mL/min, and an injection volume of 10 μL.

A Dex calibration curve was established, where *y* represents the HPLC peak area (mAU·s) and *x* represents the Dex concentration (μg/mL):

*y* = 41.045*x* + 7.5733 (R^2^ = 0.9999)

All the samples were measured in triplicate. Based on the HPLC results and the calibration curve, the Dex content was determined to be 21.9 ± 0.1 wt% for Prodrug **1** and 29.8 ± 0.2 wt% for Prodrug **2**.

**Micelle characterization**: The hydrodynamic diameter (D_h_) and PDIs of micelles were determined with a Malvern Zetasizer Nano-ZS at a 90° angle in triplicate. The samples were prepared by dissolving the polymers in deionized (DI) water at a concentration of 10 mg/mL. For DLS measurements, the aqueous solution of Prodrug **1** sample (10 mg/mL), and the supernatant of the Prodrug **2** sample (2.6 mg/mL, measured from the residual after supernatant lyophilization), were respectively sampled 20 µL and diluted by 450 µL DI water, then placed in a polystyrene semi micro cuvette, the cuvette was inserted in the chamber of Zetasizer and tested at different temperatures. Data were analyzed by the Zetasizer software version 7.12.

**In vitro drug release**: To conduct the drug release experiment, three formulations of Prodrug **2** at 25 *w*/*v*% in water were prepared and preheated to 37 °C. Releasing buffers (1.5 mL) were prepared at pH 5.0, pH 6.4, and pH 7.4 to mimic pathological acidosis and the neutral physiological environment, respectively. Sodium azide (0.01 *w*/*v*%) was added as a preservative, and DMSO (10 *v*/*v*%) was included to maintain sink conditions. These buffers were also preheated to 37 °C before being carefully layered onto the X-PEG5k-Dex_8_ formulations. Throughout the release study, the hydrogels remained intact at the bottom of the tubes without detachment, while the system was gently agitated (60 r/min) in an incubator at 37 °C. At predetermined time points, 150 μL of the supernatant was withdrawn and replaced with an equal volume of fresh buffer. Each sample was then mixed with methanol (1:1 *v*/*v*) and measured in triplicate by HPLC to quantify released Dex.

**Monoiodoacetate (MIA)-induced osteoarthritis model**: C57BL/6 mice (8 weeks old) from the Jackson Laboratory were used for the MIA model. The MIA model was established as reported previously [25]. Briefly, under anesthesia, mice received monoiodoacetate (MIA, 0.5 mg/10 μL per mice) *via* IA injection into the right knee joint. For better dispersion of the MIA, knee joints were manually extended and flexed for 30 s. The MIA model was established on the following day post-MIA injection. The mice were then randomly assigned into the following treatment groups (10 mice per group): 1. X-PEG-Dex treated group (22.7 *w*/*v*% in saline + 10% ethanol, dose equivalent of Dex = 20 mg/kg, 296 μL/kg); 2. Free Dex treated group (dexamethasone sodium phosphate 92.7 mg/mL in saline + 10% ethanol, Dex equivalent dose: 20 mg/kg, 296 μL/kg); and 3. Saline + 10% ethanol treated group (296 μL/kg). Additional mice were used as healthy controls. The incapacitance test was performed to assess the pain-related behaviors.

**Pain behavior assessment**: To test the effect of IA X-PEG-Dex treatment on amelioration of arthritic joint pain, the static weight distribution between the hind limbs of mice was measured using an incapacitance meter (Ugo Basile S.R.L., Gemonio VA, Italy), which consists of two force transducers capable of measuring the body weight that the animal places on each hind limb. Animals were placed on the apparatus with their hind paws centered on the two force transducers, and the average body weight distribution in grams was recorded. The weight-bearing score was expressed as a ratio of the weight placed through the ipsilateral limb *versus* the sum of the weights placed through both the contralateral and ipsilateral limbs, with a ratio of 50% resulting from equal weight distribution across both hind limbs. Weight distribution was measured before model establishment, before drug administration, and daily after the initiation of the treatment. All in vivo experiments were approved by the Institutional Animal Care and Use Committee (IACUC) of the University of Nebraska Medical Center, performed in accordance with the IACUC-approved protocol 25-045-00-FC.

**Statistical analysis**: Two-way analysis of variance (ANOVA), followed by Tukey’s *post hoc* test to account for multiple comparisons, was used in data analysis using GraphPad Prism 8. *p*-values ≤ 0.05 were considered as statistically significant.

## 3. Results and Discussions

### 3.1. Synthesis and Characterization of X-PEG-Dex Polymeric Prodrugs

**Scheme 1:**
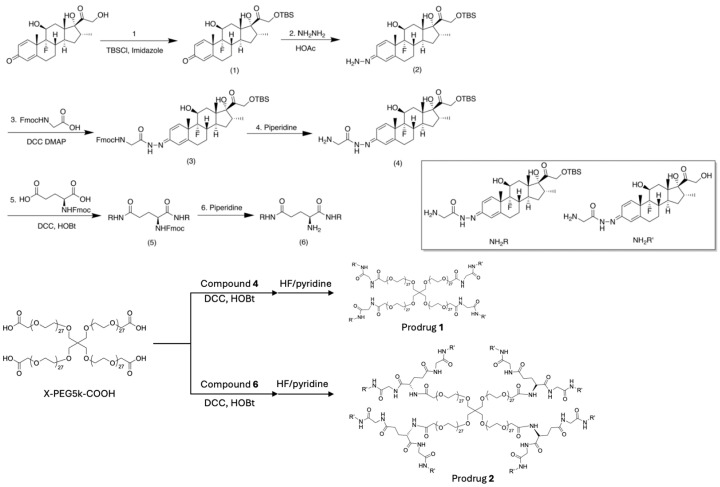
Synthetic routes of X-PEG-Dex prodrugs Prodrug **1** and **2**, via carboxylic acid–functionalized four-arm PEG intermediate (X-PEG-COOH) coupling with Dex and hydrazone-containing intermediates (Compound **4** and **6**).

Two Dex polymeric prodrugs with different Dex loadings were synthesized using a four-arm PEG scaffold (5 kDa) as the central platform. The synthetic strategy is illustrated in Scheme 1 and was adapted from our previously reported Dex prodrug chemistry [31,32].

Briefly, the carboxylic acid–functionalized four-arm PEG intermediate (X-PEG-COOH) was coupled with Dex and hydrazone-containing intermediates, via *N*,*N*’-dicyclohexylcarbodiimide (DCC)-mediated condensation. For Prodrug **1** (X-PEG5k-Dex_4_), X-PEG-COOH was reacted with Compound **4** via DCC-mediated condensation. PEG conjugate was obtained bearing the *tert*-butyldimethylsilyl (TBDMS)-protected Dex moieties. Subsequent HF/pyridine treatment removed the TBDMS protecting groups to yield the final polymeric prodrug containing four Dex units per PEG molecule. To increase the hydrophobic drug content, Prodrug **2** (X-PEG5k-Dex_8_) was synthesized using a Dex dimer intermediate (Compound **6**) as the coupling partner. In this case, X-PEG-COOH was reacted with the Dex dimer through the same DCC-mediated condensation, producing a protected PEG–Dex prodrug in which each PEG arm carries two Dex units. After HF/pyridine-mediated removal of the TBDMS protecting groups, the final polymeric prodrug containing eight Dex moieties per PEG molecule was obtained. Both polymeric prodrugs were purified by size-exclusion chromatography on a Sephadex LH-20 column to remove small-molecule impurities and unreacted intermediates. The structures of the purified polymeric prodrugs were confirmed by ^1^H NMR spectroscopy (Figure S1) and GPC (Figure S2 and Table S1).

The Dex content of the polymeric prodrugs was further quantified by HPLC analysis after acid-catalyzed full hydrolysis. The measured drug loadings, 21.9 *w*/*w*% for Prodrug **1** was consistent with the designed structures (theoretical drug loadings: 21.8 *w*/*w*%), confirming the successful synthesis of X-PEG5k-Dex_4_. The measured Dex loading of Prodrug **2** (29.8 *w*/*w*%) was slightly lower than the theoretical value (32.7 *w*/*w*%), corresponding to an average substitution degree of approximately 7.3 Dex molecules per PEG molecule. The lower loading observed for Prodrug **2** is likely attributable to incomplete coupling during the later stages of conjugation, where increasing steric hindrance associated with the bulkier Dex-containing substituents reduces the accessibility of the remaining reactive sites. Increasing the number of Dex moieties from four to eight substantially enhanced the hydrophobicity of the polymeric prodrug, which was expected to influence intermolecular hydrophobic interaction and their thermoresponsive self-assembly behavior in aqueous media [25,33,34,35,36].

### 3.2. Thermoresponsive Behavior of Prodrug ***1*** and Prodrug ***2***

**Figure 1:**
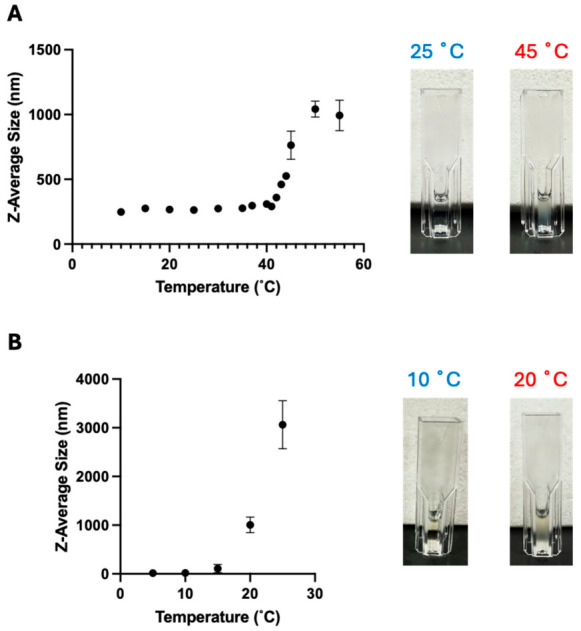
Particle sizes of (**A**) Prodrug **1** and (**B**) **2** aqueous solutions as a function of temperature variation, with representative appearances below (blue) and above (red) the transition temperature. Polymers were dissolved in deionized water at 10 mg/mL. Prodrug **1** was fully dissolved, and the sample was taken as is; Prodrug **2** was partially dissolved, and the sample was taken from the supernatant, with a measured concentration of 2.6 mg/mL, calculated from the residual after supernatant lyophilization.

To investigate the thermoresponsive aggregation behavior of the PEG–Dex polymeric prodrugs in aqueous solution, Prodrug **1** and Prodrug **2** were first analyzed at a concentration of 10 mg/mL in water using dynamic light scattering (DLS). Upon hydration at 4 °C, Prodrug **1** completely solubilized, while Prodrug **2** showed partial precipitation, indicating lower water solubility due to its higher Dex content. For DLS measurements, the supernatant of the Prodrug **2** sample, with a measured concentration of 2.6 mg/mL, was used for analysis. Both polymers exhibited temperature-dependent self-assembly behavior. As shown by the changes in Z-average hydrodynamic diameter (Figure 1), the apparent clear-to-turbid transition temperature was measured at approximately 42 °C for Prodrug **1** and 20 °C for Prodrug **2**. Below these temperatures, the solutions remained optically clear. When the temperature exceeded the transition temperature, the samples gradually became turbid, indicating increased intermolecular aggregation of hydrophobic Dex domains (Figure 1 and Figure S3) [37,38]. Notably, Prodrug **2** displayed a substantially lower transition temperature than Prodrug **1**. This behavior may be attributed to its higher hydrophobic Dex content, which promotes stronger hydrophobic Dex interactions and facilitates its thermoresponsive aggregation at lower temperatures [25,39].

To further evaluate whether these molecular aggregations could lead to macroscopic gel formation, the two polymeric prodrugs were subsequently investigated at higher concentrations, where intermolecular interactions are expected to be higher. Prodrug **1** readily formed homogeneous aqueous solutions due to its relatively low hydrophobic content (Figure S4). When dissolved in water at 30 *w*/*v*%, the prodrug produced a moderately viscous but still flowable solution. Increasing the temperature from 28 to 50 °C did not induce noticeable changes in viscosity or phase transition. The solutions remained homogeneous without sol–gel transition, even at higher concentrations (40 and 50 *w*/*v*%), suggesting that Prodrug **1** cannot produce thermoresponsive gelation. In contrast, Prodrug **2** exhibited markedly different phase transition behavior. When dissolved in water at 25 *w*/*v*%, Prodrug **2** formed a hydrogel at 4 °C. Upon warming from 5 °C to 20 °C, the hydrogel underwent a clear-to-turbid transition at approximately 15 °C (Figure S5), indicating enhanced temperature-induced polymer aggregation within the gel network.

While Prodrug **2** could form hydrogel, its viscosity was high, limiting its injectability. As an attempt to modulate Prodrug **2**’s rheological properties and make it injectable, a mixed formulation was prepared by mixing Prodrug **1** and Prodrug **2** at a weight ratio of 1:2, at an overall polymer concentration of 20 *w*/*v*%. The mixture exhibited thermoresponsive behavior, appearing as a clear light-yellow liquid at lower temperatures and becoming turbid above 30 °C. However, this transition was not accompanied by gel formation, and the sample remained a viscous but flowable liquid, as confirmed by the vial inversion method (Figure S6) [40,41].

These results indicate that increasing Dex density substantially enhances hydrophobic interactions and temperature-dependent Dex aggregation at the molecular level [25,42]. However, the balance between hydrophilic PEG segments and hydrophobic Dex domains ultimately determines whether the system forms a stable hydrogel network or remains as a viscous solution. In the future, we may further titrate the lipophilicity balance by adjusting the arm length of X-PEG, depending on its commercial availability.

### 3.3. Injectable Hydrogel Formulation of Prodrug ***2***

Although Prodrug **2** formed a thermoresponsive hydrogel, its high viscosity limited direct injection through standard syringes. To improve injectability, ethanol was introduced as a cosolvent. Prodrug **2** was first prepared at 25 *w*/*v*% in water, followed by the addition of ethanol to achieve a final formulation containing 22.7 *w*/*v*% polymer in 10% (*v*/*v*) ethanol–water. The presence of ethanol significantly reduced the viscosity of the system, resulting in a flowable formulation suitable for syringe injection [43,44,45]. The injectability of this formulation was evaluated using an insulin syringe with a 32-gauge needle. The formulation could be smoothly injected into saline without noticeable resistance. Upon injection, the solution rapidly formed a hydrogel depot (Video S1), likely due to the diffusion of ethanol into the surrounding aqueous environment, which promoted polymer aggregation and the formation of a physically crosslinked network [44,45]. This in situ gelation behavior indicates that the ethanol-modulated formulation enables facile injection while allowing the polymeric prodrug to form a hydrogel after administration, making it suitable for localized and sustained drug delivery applications.

Prodrug **2** hydrogel’s activation and Dex release kinetics were evaluated at different pH values to assess its responsiveness to pathological environments [25,27]. Prodrug **2**-based hydrogel (25 *w*/*v*% in water) was prepared at 37 °C, after which releasing buffers with pH values of 5.0, 6.4, and 7.4 were layered onto the gels to mimic acidic pathological and neutral physiological conditions. DMSO (10 *v/v*%) was included in the buffers to maintain sink conditions. Throughout the experiment, the hydrogels remained structurally stable and attached to the bottom of the tubes, indicating that the polymer network remained intact during incubation. At predetermined time points, aliquots of the supernatant were withdrawn and analyzed by HPLC to quantify the released Dex.

**Figure 2:**
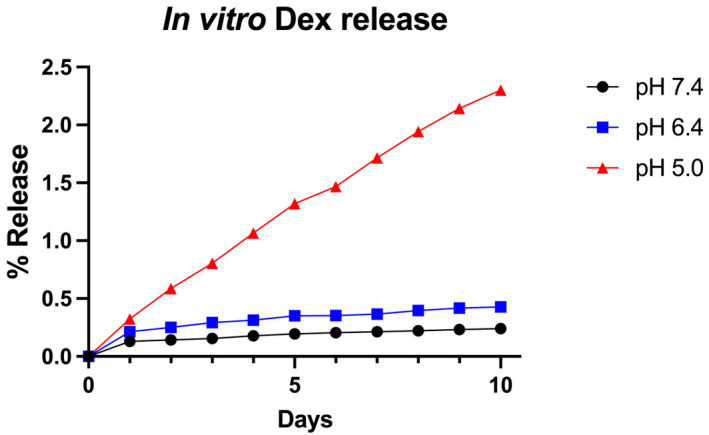
In vitro release kinetics of the percentage of Dex released from X-PEG-Dex. Hydrogels of Prodrug **2** (25 *w*/*v*% in water) were placed at the bottom of vials, and releasing buffers (pH 7.4, 6.4, and 5.0) were carefully layered on top. DMSO (10 *v*/*v*%) was included to maintain sink conditions. Vials were incubated in a 37 °C water bath, and samples were collected daily and analyzed in triplicate by HPLC. Data are presented as mean percentage of the total Dex loaded in Prodrug **2**.

As shown in Figure 2, Dex release from the hydrogel exhibited a clear pH dependence. Acidic conditions (pH 5.0) significantly accelerated the release rate, resulting in more than 2% of the total drug content after 9 days, whereas release was substantially slower at pH 6.4 and 7.4. This behavior is consistent with the acid-labile hydrazone linkage between Dex and the polymer backbone, which undergoes accelerated hydrolysis under acidic conditions. Notably, the release profile observed at pH 5.0 is comparable to previously reported results for ProGel-Dex [25], suggesting that acidic microenvironments may promote prodrug activation and enhance local drug availability in pathological tissues. 

### 3.4. Analgesic Efficacy in the MIA-Induced Osteoarthritis Pain Model

The therapeutic efficacy of the injectable hydrogel formulation was evaluated using the MIA-OA pain model, which partially reproduces key pathological features of human OA, including progressive joint degeneration and persistent pain behavior. The Prodrug **2** hydrogel formulation (22.7 *w*/*v*% Prodrug **2** in saline containing 10% ethanol) was administered via IA injection one day after MIA induction.

**Figure 3:**
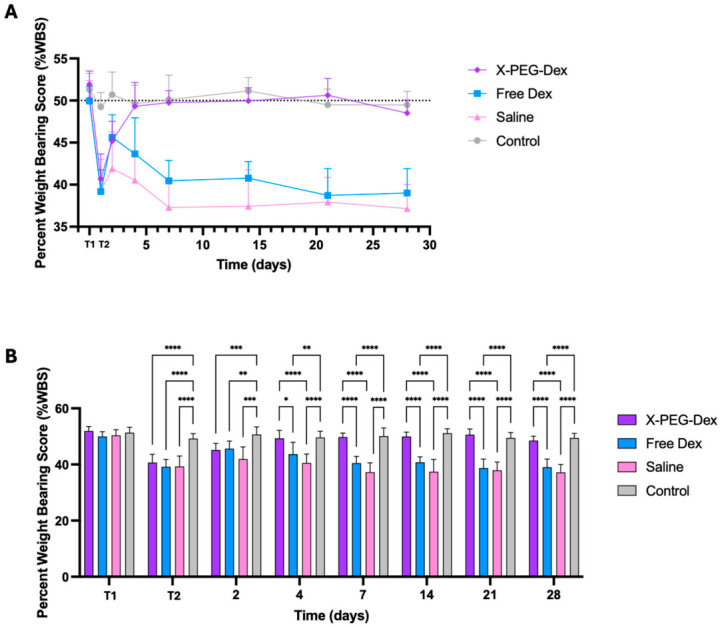
Single IA injection of X-PEG-Dex (Prodrug **2**) provided sustained joint pain relief in the monosodium iodoacetate (MIA)-induced osteoarthritis (OA) pain mouse model. (**A**) Time course of static hind limb weight distribution measured by the incapacitance test. The MIA model was established one day after MIA injection, after which mice were randomly assigned to treatment groups (*n* = 10 per group) and received IA injection of X-PEG-Dex (Dex equivalent dose: 20 mg/kg), dexamethasone sodium phosphate (free Dex, dose-equivalent: 20 mg/kg), or saline. Healthy mice without treatment were included as controls. The weight-bearing score was calculated as the ratio of the weight placed on the ipsilateral limb to the total weight on both hind limbs, with 50% (dash line) indicating equal weight distribution. (**B**) Static hind limb weight distribution at selected time points. Data are presented as mean ± SEM. Two-way analysis of variance (ANOVA) was used for inter-group comparison. *, *p* ≤ 0.05; **, *p* ≤ 0.01; ***, *p* ≤ 0.001; ****, *p* ≤ 0.0001. T1 = pre-induction; T2 = pre-injection.

The incapacitance test was performed to assess pain-related behavior, with static weight distribution between the two hind limbs measured as an indicator of joint discomfort/pain. As shown in Figure 3, animals treated with the Prodrug **2** hydrogel exhibited a rapid reduction in OA-like pain behavior, with significant improvement observed within 2 days after injection. Importantly, the analgesic effect was sustained throughout the 28-day study period, indicating prolonged therapeutic activity of the hydrogel formulation. In contrast, animals treated with dose-equivalent free Dex showed only transient improvement, with an increase in percent weight-bearing score (%WBS) on day 1 post-injection, followed by a gradual decline over time. By day 21, %WBS in the free Dex group had returned to levels comparable to those of the saline group. Overall, neither the free Dex nor saline group showed sustained improvement in weight-bearing asymmetry, suggesting that the prolonged analgesic effect observed with X-PEG-Dex (Prodrug **2**) is attributable to localized and sustained release of Dex from the hydrogel depot. No adverse behavioral effects or abnormal weight loss were observed in treated mice during the study period.

Compared with the analgesic effects revealed by the in vivo study, the overall extent of Dex release observed in vitro was relatively low over the study period. This observation can be attributed, in part, to the experimental setup of the in vitro release system [46]. In the current design, the hydrogel remained localized at the bottom of the tube, with drug diffusion occurring primarily across the gel–medium interface at the top of the gel, thereby limiting the effective diffusion area. As a result, the measured release likely underestimates the intrinsic release potential of the system. In addition, although sink conditions were approximated by the inclusion of DMSO (10 *v*/*v*%) in the release medium, they do not fully reproduce the physiological environment of the synovial cavity. Previous studies have shown that simulated synovial fluid containing physiological components, such as proteins and hyaluronic acid, provide a more representative release environment for intra-articular depot formulations [47]. Moreover, the abundant in vivo presence of protein carriers (e.g., albumin) may also facilitate the X-PEG-Dex activation equilibrium, favoring Dex release. Additionally, the static in vitro release setup does not fully recapitulate the physiological environment of the joint cavity. The continuous fluid exchange and joint articulation would facilitate hydrogel break up and erosion, thereby increasing the exposed surface area available for drug release and enhancing mass transport [48,49]. Furthermore, the rich enzymatic presence in the joint space, particularly under inflammatory conditions, may also accelerate X-PEG-Dex activation and indirectly promote drug release [50]. These observations are consistent with previous reports demonstrating enhanced drug release from enzyme-responsive depot systems under physiologically relevant conditions [51].

Importantly, previous studies from our laboratory have demonstrated that ProGel-Dex, the closely related thermoresponsive dexamethasone prodrugs, can achieve sustained therapeutic efficacy following a single intra-articular injection. In a murine osteoarthritis model, prolonged pain relief was maintained for up to 15 weeks despite similarly slow in vitro release profiles [28]. Furthermore, pharmacokinetic and biodistribution studies of the related ProGel-Dex system demonstrated prolonged intra-articular retention, sustained local drug availability, and minimal systemic exposure following a single injection [30]. Similar discrepancies between static in vitro release and in vivo drug release have also been reported for other injectable dexamethasone hydrogel systems, in which physiological exposure, mechanical effects, and enzymatic degradation resulted in substantially faster drug release in vivo than under conventional in vitro conditions [49]. It should also be noted that Dex release from the system is majorly governed by the acid-labile hydrazone linkage, and thus proceeds via a hydrolysis-controlled mechanism rather than simple diffusion from the bulk of the hydrogel. Taken together, these factors indicate that the observed in vitro release profile likely represents a rather conservative estimate, whereas the sustained analgesic efficacy observed in vivo supports effective Dex availability at the target site. Nevertheless, pharmacokinetic and biodistribution studies were not performed for the present X-PEG-Dex formulation, and future studies will focus on establishing a quantitative correlation between intra-articular drug exposure and therapeutic efficacy while developing more physiologically relevant in vitro release models.

## 4. Conclusions

In this study, two PEG-based dexamethasone polymeric prodrugs with different drug contents were developed using a four-arm PEG scaffold. Prodrug **1**, containing four Dex molecules, exhibited high aqueous solubility but did not undergo thermoresponsive gelation. In contrast, Prodrug **2**, bearing eight Dex, exhibited pronounced thermoresponsive aggregation and formed hydrogels in aqueous media driven by enhanced hydrophobic intermolecular interactions. The viscosity of the Prodrug **2** hydrogel was successfully modulated by introducing ethanol as a cosolvent, enabling the formation of an injectable formulation that rapidly forms a hydrogel depot upon exposure to an aqueous environment.

The hydrogel demonstrated pH-responsive Dex release mediated by an acid-triggered hydrazone bond cleavage, with accelerated drug release under acidic conditions. In a MIA-induced OA pain model, intra-articular injection of the Prodrug **2** hydrogel formulation produced rapid and sustained analgesic effects lasting up to 28 days, whereas free Dex showed no significant therapeutic benefit. These results show that the thermoresponsive X-PEG-Dex prodrug hydrogel enables localized and prolonged corticosteroid delivery and may represent a promising strategy for the treatment of joint pain associated with osteoarthritis and other local inflammatory pathologies.

## Data Availability

The original contributions presented in this study are included in the article/Supplementary Material. Further inquiries can be directed to the corresponding author.

## Supplementary Materials

The following supporting information can be downloaded at: https://www.mdpi.com/article/10.3390/nano16140893/s1, Figure S1: ^1^H NMR spectra of Prodrug **1** and Prodrug **2**; Figure S2: GPC traces of X-PEG5k-COOH, Prodrug **1**, and Prodrug **2**; Figure S3: Number-weighted DLS size distributions of Prodrug aqueous solutions: Prodrug **1** at 25 °C and 45 °C; Prodrug **2** at 5 °C and 15 °C; Figure S4: The appearances of Prodrug **1** in DI water at 30 *w*/*v*%, at different temperatures from 28 °C to 50 °C; Figure S5: The appearances hydrogel formed by dissolving Prodrug **2** in DI water at 25 *w*/*v*%, respectively at 5 °C and 20 °C; Figure S6: The appearance of 20 *w*/*v*% aqueous mixture of Prodrug **1** and Prodrug **2** (1:2, *w*/*w*) in response to temperature variation; Table S1: Molecular weight information of Prodrug **1** and Prodrug **2**. Video S1: In situ formation of hydrogel depot by injecting solution of Prodrug **2** in 10% (*v*/*v*) ethanol–water (22.7 *w*/*v*%) into saline.
