# Supplementary material for: The Development of Four-Arm PEG-Based Thermoresponsive Dexamethasone Prodrugs for the Treatment of Osteoarthritis Pain
Source: Nanomaterials (Basel). 2026 Jul 21;16(14):893. doi: 10.3390/nano16140893 (PMC13415126; doi:10.3390/nano16140893)
Supplement: Supplementary file 1 [file nanomaterials-16-00893-s001.zip › X-PEG-Dex-Paper-SI-Nanomaterials.v6.pdf]

The Development of Four-arm PEG-based Thermoresponsive Prodrugs of Dexamethasone for the Treatment of Osteoarthritis Pain

Yangwei Deng[1], Shabnam Arash[1], Jie Rong[1], Salma Abdullah Althobaiti[1], Shanshan Liu[1], Eleanor Ransdell-Green[1], Edward V. Fehringer[2], Dong Wang[1,2,*]

Supporting Information

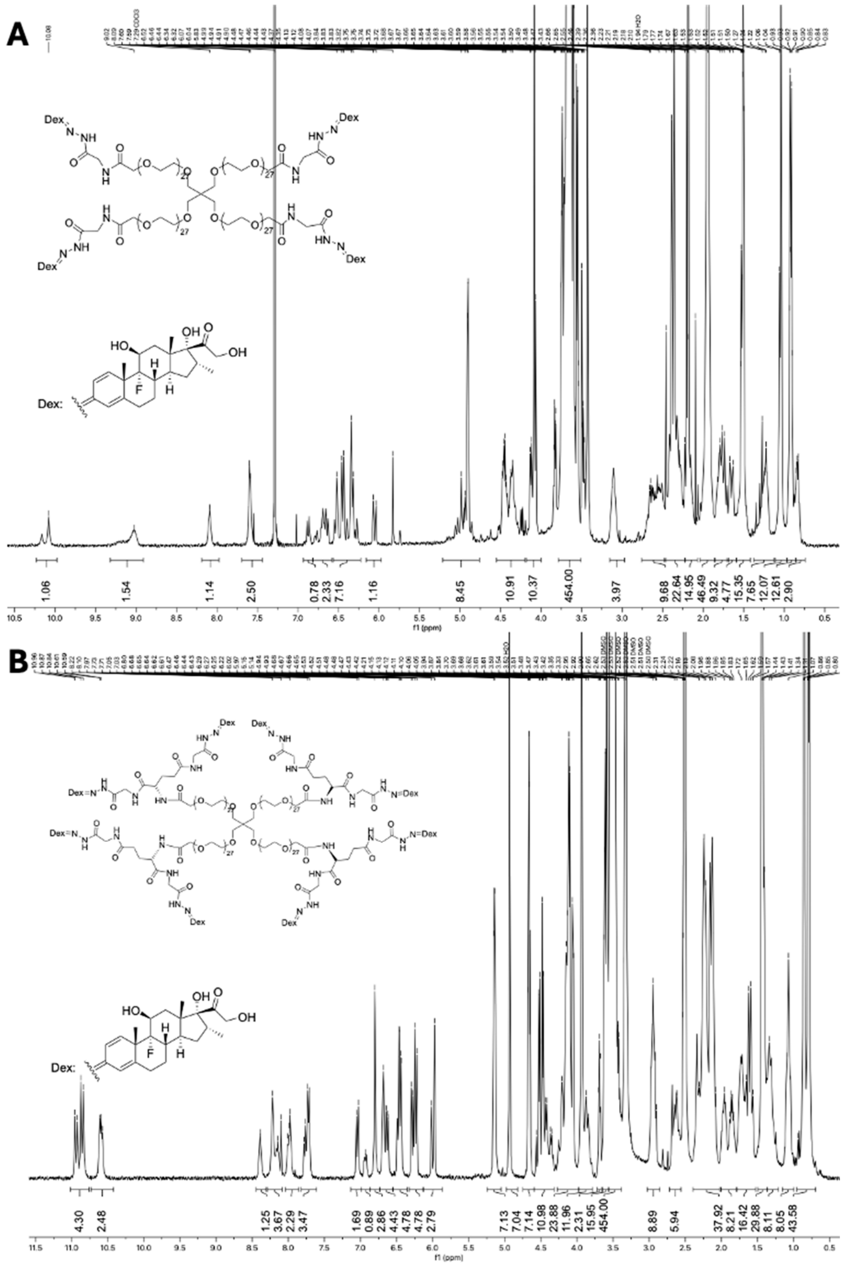

Figure S1. ^{1}H NMR spectra of (A) Prodrug **1** in $CDCl_3$ and (B) Prodrug **2** in DMSO-d_6.

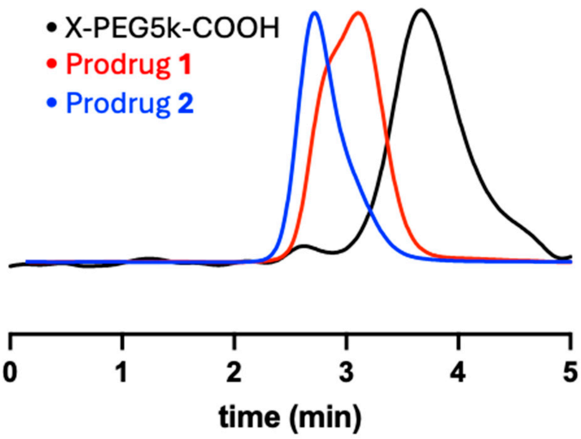

Figure S2. GPC traces of X-PEG5k-COOH, Prodrug **1**, and Prodrug **2**.

Table S1. Molecular weight information of Prodrug **1** and Prodrug **2**, including the number-average molecular weight (M_n), weight-average molecular weight (M_w), and polydispersity index (Đ).

**Polymer**	**M_n (kDa)**	**M_w (kDa)**	**Đ**
**Prodrug 1**	6.5	7.6	1.18
**Prodrug 2**	9.2	14.2	1.54

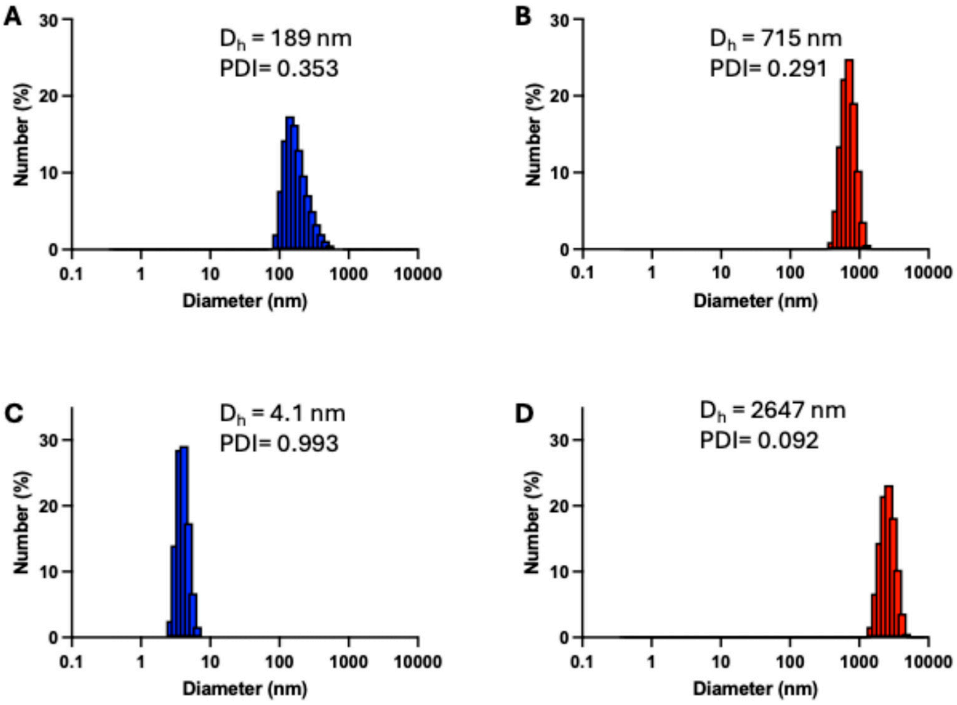

Figure S3. Number-weighted DLS size distributions of Prodrug aqueous solutions: Prodrug **1** at (A) 25 ˚C and (B) 45 ˚C; Prodrug **2** at (C) 5 ˚C and (D) 15 ˚C. The number mean hydrodynamic diameter (D_h) and polydispersity index (PDI) are presented alongside the corresponding graphs. Polymers were dissolved in deionized water at 10 mg/mL. Prodrug **1** was fully dissolved, and the sample was taken as is; Prodrug **2** was partially dissolved, and the sample was taken from the supernatant, with a measured concentration of 2.6 mg/mL, calculated from the residual after supernatant lyophilization.

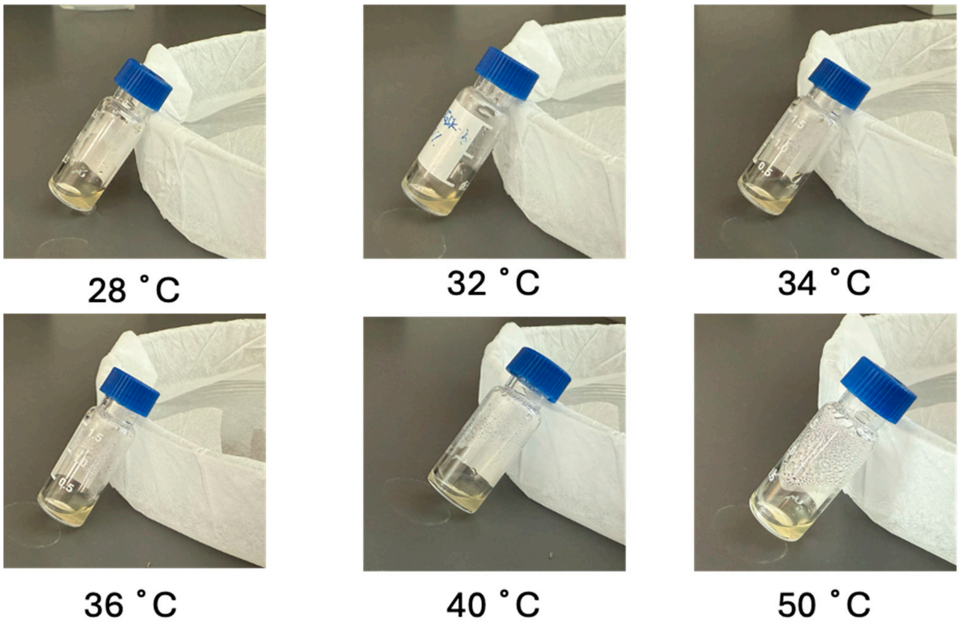

Figure S4. The appearances of Prodrug **1** in DI water at 30 w/v%, at different temperatures.

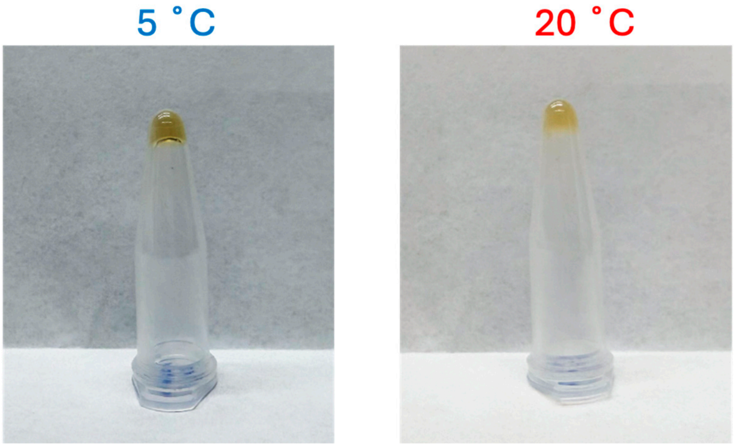

Figure S5. The appearances hydrogel formed by dissolving Prodrug **2** in DI water at 25 w/v%, respectively at 5 ˚C and 20 ˚C.

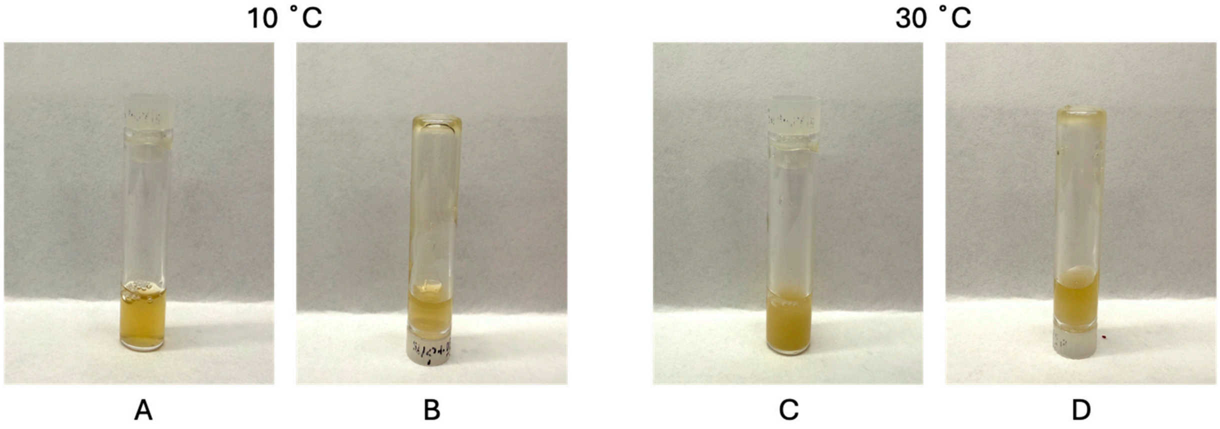

Figure S6. The appearance of 20 w/v% aqueous mixture of Prodrug **1** and Prodrug **2** (1:2, w/w) in response to temperature variation.
